# Role of CD_4_^+^ T and CD_8_^+^ T Lymphocytes-Mediated Cellular Immunity in Pathogenesis of Chronic Obstructive Pulmonary Disease

**DOI:** 10.1155/2022/1429213

**Published:** 2022-06-22

**Authors:** Weilin Xue, Jianying Ma, Yue Li, Chunxia Xie

**Affiliations:** ^1^Department of Respiratory Medicine, Qingdao Hiser Medical Center, Qingdao, 266033 Shandong Province, China; ^2^Department of General Medicine, Qingdao Hiser Medical Center, Qingdao, 266033 Shandong Province, China

## Abstract

This work was to explore the changes of T lymphocyte subsets in peripheral blood of patients with acute exacerbation of chronic obstructive pulmonary disease (COPD) (AECOPD) and the role of cellular immunity mediated in the disease process. Eighty-six patients with AECOPD who visited Qingdao Hiser Medical Center from June 2020 to December 2021 and 30 healthy people (controls) who underwent health examination in the same period were selected. The differences of pulmonary function (PF), arterial blood gas (ABG), blood routine inflammatory indexes, T lymphocyte and T lymphocyte subsets were compared between the two groups, and the correlation between T lymphocyte subsets and each index was analyzed. There were clear differences in PF, ABG, and PB inflammation indexes between AECOPD patients and the controls (*P <*0.05). Compared with the controls, the CD_4_^+^ and CD_4_^+^/CD_8_^+^ ratio in PB of AECOPD group were obviously decreased, and the CD_8_^+^ level was clearly increased (*P <*0.05); Th1 of CD_4_^+^ cell subsets and Tc1 of CD_8_^+^ cell subsets were significantly increased, while Th2 of CD_4_^+^ cell subsets and Tc2 of CD_8_^+^ cell subsets were obviously decreased (*P <*0.05). However, CD4^+^ was significantly positively correlated with lung function indexes, and significantly negatively correlated with neutrophils/lymphocytes and high-sensitivity C-reactive protein (*P* <0.05) and significantly positively correlated with Hs-CRP (*P <*0.05). In summary, CD4+ and CD8^+^ T lymphocytes were involved in the occurrence and occurrence of AECOPD, the decrease of CD4^+^ and the increase of CD8^+^ may promote the deterioration of COPD.

## 1. Introduction

COPD is an airway inflammatory disease characterized by airflow limitation, complete irreversible, and continuous progressive development [1]. Statistics show that the incidence of COPD is 1%, but the incidence of people older than 40 years shows a sharp increase [2]. COPD patients continue to deteriorate in a short period of time, and are accompanied by significant aggravation such as fever, which manifests as acute exacerbation of COPD (AECOPD). At this time, it is necessary to change the routine medication of COPD [3]. At present, there is no specific drug for the treatment of COPD, so the main purpose in clinical practice is only to relieve the symptoms of patients. Measurement of PF is the gold standard for the diagnosis of COPD, but patients in acute exacerbation need to be combined with other biomarkers for comprehensive diagnosis [4]. Inflammatory indicators in blood routine have certain value for the diagnosis of COPD, evaluation of therapeutic effect, and evaluation of patient prognosis, such as white blood cell (WBC) count, NLR, platelet-lymphocyte ratio [5, 6].

The pathogenesis of COPD is very complex, and it is generally accepted that the main features of COPD are chronic inflammatory responses in the airways, lung parenchyma, and pulmonary vessels [7]. Studies have confirmed that COPD belongs to a mixed immune response, and the immune response of this disease includes innate and adaptive immunity, and macrophages, neutrophils, and T lymphocytes are involved [8, 9]. Macrophages are ubiquitous in the airways and lung soft tissues of COPD patients, and smoking can activate this cell to release inflammatory mediators, which in turn accelerate the process of the disease [10]. Cellular immunity is involved in the progression of COPD disease. Neutrophil wiping bathed in AECOPD integration and is strongly associated with pulmonary failure in patients [11]. T lymphocytes are the main inflammatory cells in the central airway and lung parenchyma of COPD patients, which are related to the number of alveolar injuries and the degree of airway obstruction in patients [12]. It was found that the main type of CD_4_^+^ T lymphocytes accumulated in the airways and lung tissues of COPD patients is Th1, which can cause the destruction of lung tissues and lead to emphysema by secreting inflammatory cytokines and chemokines [13]. The increase of CD_8_^+^ T lymphocytes is the main feature of T lymphocyte infiltration into the airways and lungs, and CD_8_^+^ T lymphocytes are closely related to airway limitation as well as the occurrence and development of the disease [14].

This work was to further explore the mechanism of cellular immunity mediated by T lymphocyte subsets in the occurrence and development of COPD and to improve the therapeutic effect and prognosis of COPD patients. The changes of PF, ABG, PB inflammation, and T lymphocyte subsets in patients with AECOPD were compared and analyzed to understand the disorder of immune function in COPD patients, and to provide research ideas for the clinical diagnosis and immunotherapy of AECOPD.

## 2. Materials and Methods

### 2.1. Research Objects

A total of 86 patients with AECOPD diagnosed in the Department of Respiratory Medicine of our hospital from June 2020 to December 2021 were selected as the AECOPD group; 30 patients who underwent health examinations in the outpatient physical examination center of our hospital during the same period were selected as the control group. The experiment was performed according to the diagnostic and therapeutic criteria in the Global Initiative for Chronic Obstructive Lung Disease (2018GOLD). AECOPD group had 52 males and 34 females, aged 48~74 years, with mean age of (60.35 ± 4.33) years. Controls had 19 males and 11 females, aged 49~72 years, with mean age of (61.02 ± 5.49) years. All subjects understood the experimental procedures and signed the informed consent form. The experiment was approved by the medical ethics committee of Qingdao Hiser Medical Center.

Inclusion criteria: patients meeting the diagnostic criteria for AECOPD in the 2018GOLD [15]; patients with clinical manifestations including dyspnea, chronic cough, or expectoration, and/or with exposure history of COPD risk factors; patients' PF tests showed that the ratio of forced expiratory volume in the first second to forced vital capacity (FEV_1_/FVC) after inhalation of bronchodilators is less than 0.7; the deterioration of respiratory symptoms occur in a short time, such as cough, shortness of breath, wheezing, increased phlegm, requiring additional treatment or change of treatment in patients.

Exclusion criteria: patients combined with bronchial asthma, tuberculosis or pulmonary fibrosis, and other respiratory diseases; except the lungs, patients' other organs or tissues occur infection; patients are combined with cancer, bone and joint diseases or blood diseases, which can cause systemic inflammatory response disease; patients using immunosuppressive agents, non-steroidal anti-inflammatory drugs, or systemic glucocorticoids within 1 month before the examination; patients had major surgery within 3 months before the examination; patients combined with severe liver and kidney dysfunction, cardiovascular and cerebrovascular diseases, or other autoimmune diseases; patients cannot cooperate with the examiner due to cognitive dysfunction,.

In the controls, there was no history of chronic underlying diseases, no history of infection recently, and no abnormality in chest radiography in people.

### 2.2. Observation Indexes

1. Blood routine

The blood routine parameters of the subjects were measured by automatic hematology analyzer (Mindray, Nanjing Baden Medical Co., Ltd.) after treatment, including WBC, neutrophil percentage (NEU%), NLR, and eosinophil percentage (EOS%). The levels of cystin C (CysC), serum creatinine (Scr), blood urea nitrogen (BUN), and Hs-CRP were measured by immunoturbidimetry in automatic biochemical analyzer.

(2) PF

During the examination, the patient was required to take a seated position, and the medical staff carefully introduced the process of the pulmonary function examination to the patient to relieve the patient's anxiety. The patient inhaled with the greatest force, held the breath, and breathed with the greatest force and the fastest speed until the lungs were completely “emptied”. After the above actions were repeated, the patient could relax, and the breath was held for 6 s after the lungs were completely “emptied”. The related indicators of PF of the subjects were measured by pulmonary function tester (Anke, Shandong Broke Regenerative Medicine Co., Ltd.), which were FEV_1_, FVC, and FEV_1_/FVC.

(3) ABG parameters

It should measure the patient's body temperature before the examination, and select the artery according to the oxygen concentration. After sterilization of the skin, the arterial blood was automatically pushed into the blood gas needle, and the eye of the needle was blocked with a rubber stopper. Then, the blood gas needle was gently rotated to fully mix the blood and heparin, and the oxygen concentration and other indicators were calculated. ABG parameters of the subjects, including partial pressure of oxygen (PaO_2_), saturation of oxygen (SaO_2_), and partial pressure of carbon dioxide (PaCO_2_), were measured by blood gas analyzer (Model ABL90, Shanghai Radiometer Medical Equipment Co., Ltd.).

(4) Immune cell level

The venous anticoagulation was taken and centrifuged at 4,000 rpm for 10 min, the plasma was taken into the test tube, the corresponding antibody was added, PE and FITC were used to label T lymphocytes, and it was incubated at room temperature for 15 min. An appropriate amount of red blood cell lysis solution was added, it was placed in a water bath at 37°C for 20 min, phosphate buffer was added to wash the cells. After centrifugation, the immune T cell subsets CD4^+^, CD8^+^, CD4^+^/CD8^+^ were detected and analyzed by flow cytometry (CytoFLEX, China Beckman Coulter Trading Co., Ltd.). Among them, CD_4_^+^ cells secreting IFN-*γ* were Th1 cells and CD_4_^+^ cells secreting IL-4 were Th2 cells; CD_8_^+^ cells secreting IFN-*γ* were Tc1 cells and CD_8_^+^ cells secreting IL-4 were Tc2 cells.

### 2.3. Statistical Analysis

SPSS 19.0 was adopted for the collation and analysis of general data of patients and experimental data. The measured data were expressed by mean ± standard deviation ($\overset{¯}{\text{x}}$±s), and the differences were compared and analyzed using the independent sample *t*-test. Spearman correlation coefficient was adopted to detect the correlation of the indicators. *P <*0.05 indicated the differences were statistically meaningful.

## 3. Results

### 3.1. Comparison of General Data

The differences of general data of the subjects between the two groups were compared (Table 1). After comparison, it was found that there was no significant difference in gender ratio, mean age, body mass index (BMI), smoking history, and drinking history between AECOPD group and controls (*P >0.05*). Therefore, the subsequent data were comparable.

### 3.2. Changes of PF and ABG Indicators in Patients with AECOPD

**Figure 1:**
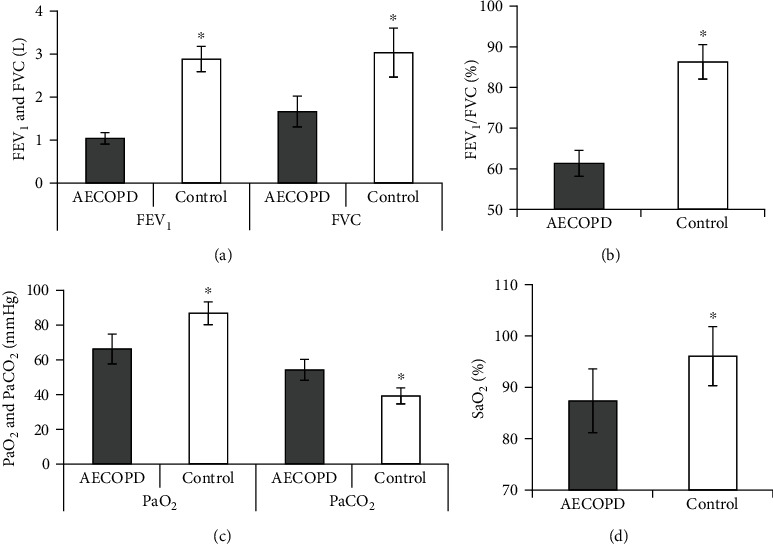
Comparison of PF and ABG parameters in patients. Note: (a) FEV_1_ and FVC; (b) FEV_1_/FVC; (c) PaO_2_ and PaCO_2_; (d) SaO_2_; in contrast with AECOPD group, the difference was statistically meaningful, ∗*P* < 0.05.

The differences in PF and ABG indicators between the two groups were compared (Figure 1). It suggested that the PF indicators FEV_1_, FVC, FEV_1_/FVC, and ABG indicators PaO_2_, SaO_2_ in the controls were distinctly superior (*P <*0.05). However, the ABG indicator PaCO_2_ in the controls was distinctly inferior (*P <*0.05).

### 3.3. Changes of Blood Routine Inflammatory Indicators in Patients with AECOPD

**Figure 2:**
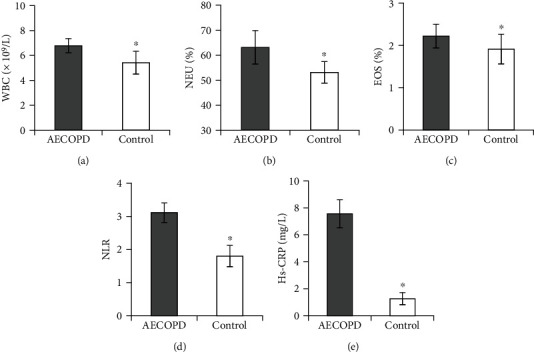
Comparison of blood routine inflammatory indicators in patients. Note: (a) WBC; (b) NEU; (c) EOS; (d) NLR; (e) Hs-CRP; there was clearly different from AECOPD group, ∗*P* < 0.05.

The differences in the levels of WBC, NEU, EOS, NLR, and Hs-CRP in blood routine between the two groups were compared (Figure 2). The levels of inflammatory indicators WBC, NEU, EOS, NLR, and Hs-CRP in the PB of the controls were clearly inferior, and the distinction had statistical meaning.

### 3.4. Changes of Renal Function Indicators in Blood Routine in Patients with AECOPD

**Figure 3:**
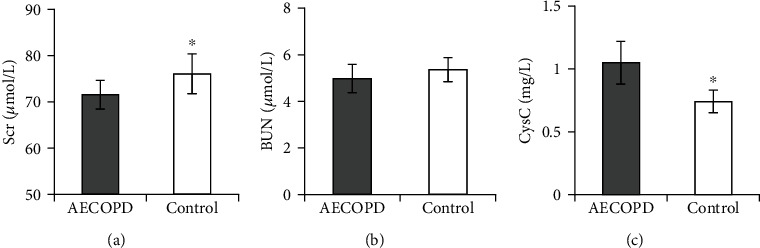
Comparison of renal function indicators in blood routine in patients. Note: (a) Scr; (b) BUN; (c) CysC; there was significantly distinct from AECOPD group, ∗*P* < 0.05.

The differences in the levels of Scr, BUN, and CysC in renal function indicators between the two groups were compared (Figure 3). The level of Scr, an indicator of renal function, in the PB of the controls was apparently superior (*P <*0.05). The CysC level in the controls was obviously lower (*P <*0.05). The difference in BUN level had no statistical significance (*P >*0.05).

### 3.5. Changes of T Lymphocyte Ratio in PB in AECOPD Patients

**Figure 4:**
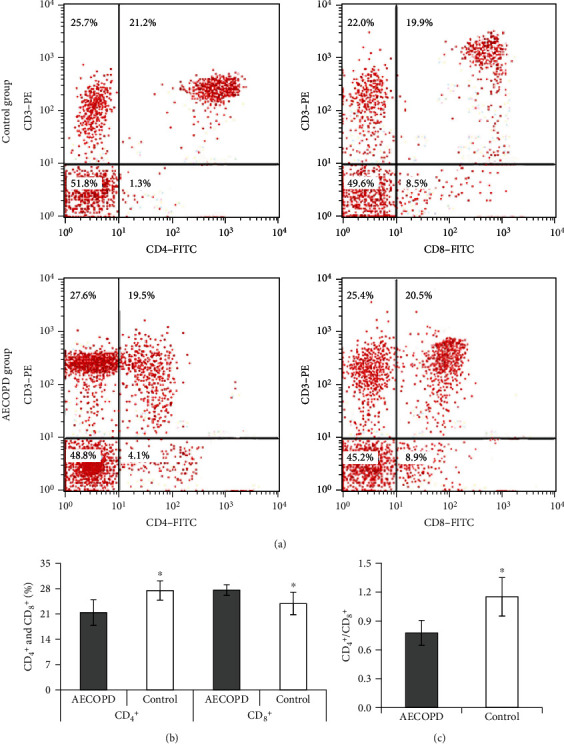
Comparison of CD_4_^+^ and CD_8_^+^ T lymphocyte levels in PB. Note: (a) CD_4_^+^ and CD_8_^+^ T lymphocyte flow cytometry; (b) CD_4_^+^ T lymphocyte level; (c) CD_8_^+^ T lymphocyte level; relative to AECOPD group, the distinction was statistically significant, ∗*P* < 0.05.

The changes of CD_4_^+^ and CD_8_^+^ T lymphocytes in PB of the two groups were detected by flow cytometry (Figure 4). After comparison, it was found that the CD_4_^+^ T lymphocyte level and CD_4_^+^/CD_8_^+^ ratio in the PB of the controls were clearly superior (*P* <0.05). The level of CD_8_^+^ T lymphocytes in PB of the controls was apparently inferior (*P* <0.05).

### 3.6. Changes of CD4+ and CD8+ T Lymphocyte Subsets Proportion in PB of AECOPD Patients

**Figure 5:**
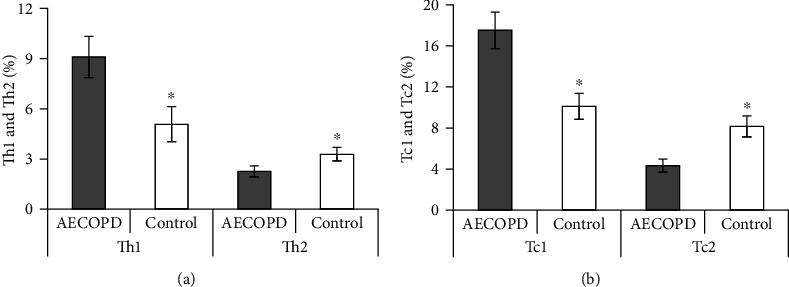
Comparison of CD_4_^+^ and CD_8_^+^ T lymphocyte subsets in PB. Note: (a) CD_4_^+^ T lymphocyte subsets level; (b) CD_8_^+^ T lymphocyte subsets level; in contrast with AECOPD group, the difference was statistically meaningful, ∗*P* < 0.05.

The differences in the proportion of CD_4_^+^ and CD_8_^+^ T lymphocyte subsets in PB between the two groups were further compared (Figure 5). The CD_4_^+^ T lymphocyte subsets Th1 and CD_8_^+^ T lymphocyte subsets Tc1 in the PB of the controls were significantly lower (*P <*0.05). The Th2 and Tc2 in PB of the controls were obviously superior than those in the AECOPD group (*P <*0.05).

### 3.7. Correlation Analysis between T Lymphocytes and Various Indicators in AECOPD Patients

The correlation between CD4, CD8, and CD4/CD8 and PF, ABG, and inflammatory indexes was compared (Table 2). CD4 was found to be apparently positively correlated with FEV_1_ and FVC, and apparently negatively correlated with NLR and Hs-CRP (*P <*0.05); CD8 and CD4/CD8 were clearly negatively correlated with FEV_1_ and FVC, significantly positively correlated with NLR and Hs-CRP (*P <*0.05); the rest indexes had some correlation with CD4, CD8, and CD4/CD8, but not significantly (*P >*0.05).

## 4. Discussion

The prevalence and mortality of COPD are increasing year by year, and it has become the third leading cause of death and the fifth leading cause of disability in the world [16]. In China, the probability of COPD disease in people over 40 years of age is as high as more than 8% [17]. While COPD is an important risk factor predisposing to lung cancer, about 50% to 80% of lung cancer patients have a history of COPD [18]. Patients with advanced COPD showed progressive reduction in PF, repeated and aggravated clinical diagnosis, and poor quality of life [19]. Therefore, it is the focus of current research to find effective drugs for the treatment of COPD and improve the prognosis of patients.

Inflammatory mediators and anti-inflammatory mediators are generated in the lungs to participate in the imbalance and lead to the disorder of the patient's respiratory system defense function. The massive release of inflammatory mediators in COPD patients causes immune imbalance, which eventually leads to hyperresponsiveness of the airway and lung tissue inflammatory response [20]. In this work, patients with AECOPD were used as the research objects, and healthy people were undertaken as controls to analyze the changes of pulmonary function, arterial blood gas, and inflammatory cell markers in peripheral blood in patients with AECOPD. The results showed that the levels of WBC, NEU%, EOS%, NLR and Hs-CRP in peripheral blood of AECOPD patients were significantly higher than those of healthy controls. WBC is a cellular marker used to evaluate the systemic non-specific inflammatory response, and WBC is closely related to the severity of COPD, and is one of the risk factors leading to reduced PF [21]. NLR can reflect the changes in the body's immune system, and it can be used in the prediction of the severity, deterioration, and mortality of COPD patients [22]. NEU % and EOS % may be involved in activating the secretion of inflammatory mediators and thus participate in the process of COPD, while phagocytosis of EOS by alveolar macrophages can reduce the ability of macrophages to clear necrotic or apoptotic cells, which is related to the number and severity of COPD exacerbations [23]. Hs-CRP is a sensitive indicator for evaluating body infection, which is significantly increased in inflammatory response, tissue damage, and immune system diseases [24]. Therefore, it is indicated that WBC, NEU%, EOS%, NLR and hs-CRP can be used as biochemical indicators for auxiliary diagnosis of AECOPD.

Oxidative stress in lung tissue and accumulation of excess protease are both important factors that cause pulmonary inflammatory response and damage in COPD patients [25]. CysC is a major extracellular cathepsin inhibitor, which has been confirmed as an inflammatory marker of acute exacerbation [26]. CysC is absorbed and metabolized by renal tubules after glomerular filtration, and the kidney is the only organ to remove CysC. Scr and BUN were combined to assess the renal function in AECOPD patients. The results revealed that the Scr level in AECOPD patients was significantly decreased, while CysC was significantly increased. It indicated that AECOPD patients had early renal damage, and CysC could be adopted as a biological indicator for evaluating renal damage in COPD patients.

The main airway lesion site in COPD is the inflammatory response of the small airways, and the main airway cytological changes are neutrophils, lymphocytes, as well as macrophages [27]. Data have shown that CD_4_^+^ and CD_8_^+^ T lymphocytes play an important role in the airway inflammatory response in COPD [28]. CD_4_^+^ T lymphocytes are accessory cells, which can release cytokines and assist other inflammatory cells in their activities in the activated state [29]. CD_8_^+^ T lymphocytes are cytotoxic cells, which are cell subsets that clear infected or damaged cells [30]. The constant CD_4_^+^/CD_8_^+^ ratio can maintain the normal immune system function in the body, and plays an important role in regulating immune balance [31]. The results indicated that CD_4_^+^ and CD_4_^+^/CD_8_^+^ ratio in PB were significantly increased, while CD_8_^+^ levels were clearly decreased in AECOPD patients. CD_4_^+^ T lymphocytes can be divided into Th1 and Th2. Th1 cells can secrete IFN-*γ* and TNF-*α*, and play a role in inducing phagocyte-mediated anti-infective immunity [32]. Th2 cells can secrete inflammatory factors such as IL-4 and IL-6 and participate in humoral immune responses [33]. Killer T cells in CD_8_^+^ T lymphocytes include Tc1 and Tc2, both of which are cytotoxic and dependent on the expression of MHC-I molecules [34]. In peripheral small airways, Tc1 cells can secrete and release IFN-*γ* to act on alveolar macrophages and attract the infiltration of neutrophils [35]. Perforin secreted by Tc1 cells combined with proteases secreted by other cells can cause alveolar tissue damage [36]. The results showed that Th1 and Tc1 were significantly higher, while Th2 and Tc2 were significantly lower in the PB of AECOPD patients. This is because Th1 cells secrete IFN-*γ* to inhibit the proliferation of Th2 cells, which aggravates the pathological damage caused by Th1-mediated protective immunity after imbalance and promotes the pulmonary inflammatory response in COPD patients. The increase in the number of Th1 cells is also able to induce the proliferation of Tc1 cells, which in turn inhibits the proliferation of Tc2 cells.

## 5. Conclusion

CD_4_^+^ and CD_4_^+^/CD_8_^+^ ratio in PB of AECOPD patients increased obviously, while CD_8_^+^ level decreased significantly. The worse the PF of AECOPD patients, the higher the level of inflammatory mediators in PB, the lower the level of CD_4_^+^ T lymphocytes in PB, and the higher the level of CD_8_^+^ T lymphocytes. In conclusion, T lymphocyte-mediated cellular immune dysfunction has an influence in the process of AECOPD, which participates in and promotes the deterioration of COPD. However, the interaction mechanism between T lymphocytes and COPD exacerbation had not been clearly elucidated. In future research, it is hoped that by preparing corresponding animal models or expanding clinical research samples, to further explore the mechanism of action of T lymphocyte subset changes on AECOPD. The results of this work are of great significance for understanding the pathogenesis of AECOPD and improving the prognosis of COPD patients.

## Figures and Tables

**Table 1:** Comparison of general data.

Information	AECOPD (n =86)	Controls (n =30)	Statistic value	*P*
Gender (males/females)	52/34	19/11	-0.319	0.314
Age (years)	60.35 ± 4.33	61.02 ± 5.49	0.433	0.326
BMI (kg/m^2^)	26.31 ± 1.26	27.03 ± 1.43	0.329	0.431
Smoking history [cases (%)]	48 (55.8)	16 (53.3)	-0.575	0.558
Drinking history [cases (%)]	32 (37.2)	9 (30.0)	-0.208	0.207

Note: BMI = [body weight (kg)]/[height ^2^(m)].

**Table 2:** Correlation analysis of CD4, CD8, and CD4/CD8 with various indicators in patients.

		CD4	CD8	CD4/CD8
PF	FEV_1_	0.257∗	-0.218∗	-0.311∗
	FVC	0.209∗	-0.208∗	-0.262∗

ABG	PaO_2_	0.094	-0.033	-0.077
	PaCO_2_	-0.028	0.024	0.035
	SaO_2_	0.105	-0.069	-0.071

Inflammatory indexes	NEU%	-0.055	0.067	0.073
	EOS%	-0.098	0.101	0.094
	NLR	-0.368∗	0.219∗	0.344∗
	Hs-CRP	-0.415∗	0.250∗	0.306∗

Note: the distinction was statistically significant, ∗*P* < 0.05.

## Data Availability

The data used to support the findings of this study are included within the article.
